# Improving the textural and microstructural quality of cow meat by black chokeberry, grape, and hawthorn vinegar‐based marination

**DOI:** 10.1002/fsn3.3566

**Published:** 2023-07-17

**Authors:** Kubra Unal, Ali Samet Babaoğlu, Mustafa Karakaya

**Affiliations:** ^1^ Department of Food Engineering, Agriculture Faculty Selçuk University Konya Turkey

**Keywords:** old carcass, tenderization, tough meat, vinegar‐based marinade

## Abstract

This study was designed to investigate the effects of vinegar‐based marinades on pH, technological properties, color, microstructure, texture, and sensory characteristics of 9‐year‐old Holstein meat which had tough texture. To marinate the *Longissimus lumborum* steaks, three different marinades were prepared depending on the different additions of vinegar: black chokeberry (BV), grape (GV), and hawthorn vinegars (HV). The group with water (without vinegar) was prepared as a control sample (C). The steak samples were dipped into the vinegar‐based marinades and stored at 4°C for 24 h. Vinegar‐based marinades decreased the pH and cooking loss of the samples (*p* < .05). The highest a* values were determined in the BV group, while the samples marinated with vinegar‐based marinades had lower L* values than the control group (*p* < .05). The samples marinated with grape vinegar marinade had the lowest hardness, WBSF, and WBSE values (*p* < .05). SEM images and sensory analysis results also supported these texture results. The results suggest that grape vinegar‐based marinade may be a promising natural tenderizer to improve textural characteristics of tough meats.

## Graphical abstract

In this study, the effects of black chokeberry, grape, and hawthorn vinegars on the textural and technological properties of old cow meat were investigated. The results suggest that grape vinegar‐based marinade may be a promising natural tenderizer to improve textural characteristics of tough meats.

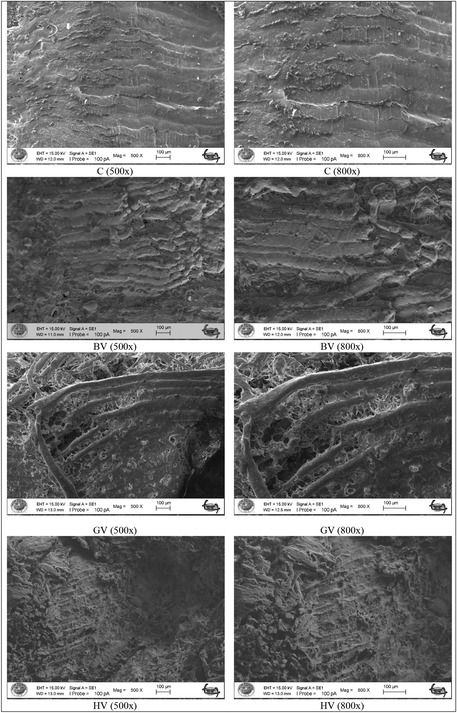

## 1 INTRODUCTION

Texture is the most important quality characteristic of meat. It determines consumer acceptance and the marketability of the final products. Texture quality is evaluated by consumers based on the sensory aspects of appearance, tenderness, and juiciness (Lee et al., 2014). Tenderness has been identified as the most important factor influencing consumer satisfaction. The tenderness of meat is influenced by the length of the sarcomeres and the structural integrity of the myofibrils, which affect actomyosin toughness. Meat from older animals is usually tougher than meat from young animals. This is due to the physicochemical nature of muscle fiber fragmentation and the more connective tissue (collagen cross‐links) of old carcasses (Han et al., 2009). As a result, the meat industry has difficulty providing consumers with meat that has consistent quality characteristics such as tenderness, juiciness, and flavor. Improving the tenderness of beef is the goal of studies being conducted by researchers around the world. One of the possible ways to tenderization of meat is marination.

Marination is traditionally used in the meat industry and is the treatment of meat with various ingredients such as fruit juices, spices, herbs, vinegar, wine, fermented dairy products, sugar, salt, oil, phosphates, acids, flavoring components, and other additives using various techniques (Roudbari et al., 2020). The effect of marination varies depending on the ingredients included in the composition of the marinade (Yusop et al., 2011). Acidic marinades increase the tenderness of meat by lowering the pH of the meat, which in turn leads to a weakening of the muscle structure, an intensification of proteolysis by cathepsins, and an increased conversion of collagen into gelatine (Sengun et al., 2021). Therefore, it is important to use vinegar in marinade recipes due to low pH of vinegars.

Fruit vinegar, made from fruits or fruit juices, has become more popular in recent years. Fruit vinegars are rich in organic acids such as acetic, tartaric, formic, lactic, citric, and malic acids, but also contain high levels of phenolic compounds, vitamins, and minerals (Bakir et al., 2017). The composition of vinegar can change depending on the raw material used and the type of production process used (Ozen et al., 2020). Grape in particular are the most commonly used fruit for vinegar production (Özdemir et al., 2022). Black chokeberry (*Aronia melanocarpa*) (Choi et al., 2016) and hawthorn (*Crataegus tanacetifolia* (lam.) pers.) fruits have also been reported to have potential for use in fruit vinegars (Kadas et al., 2014).

Several studies have reported the effectiveness of marination for improving tenderness and microbiological quality of different meats (Abdel‐Naeem et al., 2022; Babikova et al., 2020; Latoch, 2020; Lawrence & Lawrence, 2021; Sengun et al., 2019; Tkacz et al., 2021; Unal et al., 2022; Vişan et al., 2021). However, it has been observed that studies examining the effects of vinegar‐based marination on meat quality have increased in recent years (Karam et al., 2020; Mazaheri Kalahrodi et al., 2021; Sengun et al., 2021). According to the author's best knowledge, there is no study that investigates the effects of marinades with black chokeberry and hawthorn vinegar on the quality of cow meat. On the other hand, there are also no studies on the tenderization of cow meat through marinating. To fill this gap, this study was conducted to determine the effects of different marinades, including grape, black chokeberry, and hawthorn vinegars on the pH, technological properties (cooking loss, marinade uptake, drip loss, and yield), color, microstructural, textural, and sensory characteristics of *Longissimus lumborum* muscles of the cow. In addition to the meat quality analyses, the phenolic and organic acid profiles of grape, black chokeberry, and hawthorn vinegars were investigated.

## 2 MATERIALS AND METHODS

### 2.1 Materials

In the current study, the *Longissimus lumborum* muscle of three Holsteins (sex: female; age: 9 years; carcass weight: 240 ± 20 kg) reared under the same feeding and environmental conditions was collected 24 h after slaughter at a meat plant (Yilet). The muscles were cut into 2.5‐cm‐thick steaks weighing about 170 g. A total of 72 steaks were assigned randomly to four treatments (18 steaks for each treatment).

Traditionally produced black chokeberry and grape vinegars were sourced from Konya, Turkey, and hawthorn vinegar from Kastamonu, Turkey. Olive oil (Kristal), salt (Salina), and black pepper (Bagdat) were purchased from a market in Konya, Turkey.

### 2.2 Preparation of marinades

In the study, three different marinades were prepared with different vinegar additions: black chokeberry vinegar (pH: 3.75), grape vinegar (pH: 2.95), and hawthorn vinegar (pH: 3.20). A control sample was prepared with water instead of vinegar. The marinades were prepared according to our previous study with some modifications (Dilek et al., 2023). The formulation of marinades is given in Table 1. In the formulation of the marinades containing vinegars, 200 mL of water was replaced by 200 mL of the corresponding vinegar. When preparing the marinades, all the ingredients given in Table 1 were mixed for 10 min. The prepared marinades were left at room temperature for at least 2 h with constant stirring to allow the dry ingredients to hydrate.

**TABLE 1:** Formulations of marinades.

Formulation	Treatments			
	C	BV	GV	HV
Water (mL)	300.0	100.0	100.0	100.0
Black chokeberry vinegar (mL)	‐	200.0	‐	‐
Grape vinegar (mL)	‐	‐	200.0	‐
Hawthorn vinegar (mL)	‐	‐	‐	200.0
Olive oil (mL)	100.0	100.0	100.0	100.0
Salt (g)	4.0	4.0	4.0	4.0
Black pepper (g)	0.4	0.4	0.4	0.4

*Note*: C: Samples marinated with water (vinegar free‐control), BV: Samples marinated with black chokeberry vinegar, GV: Samples marinated with grape vinegar, HV: Samples marinated with hawthorn vinegar.

### 2.3 Marination and cooking procedure

The steak samples were randomly and individually dipped into the marinade solutions in plastic bags at a ratio of 1:1 (meat: marinade) and stored at +4°C for 24 h. After marination, the marinades were removed from the steak samples.

The samples were grilled at a temperature of 195°C until an internal temperature of 72°C was reached. The temperature was measured with a digital thermometer (Digitale Bratengabel‐TCM) and the samples were grilled for 4.5 min on each side.

### 2.4 Analyses in vinegars

#### 2.4.1 Determination of TPC, TFC, and DPPH values

The total phenolic content (TPC) of the vinegars was determined by the Folin–Ciocalteu method as described by Yoo et al. (2004). Absorbance was measured at 750 nm against a reagent blank in a ultraviolet–visible spectrophotometer (UV‐160 A, UV–Visible Recording Spectrophotometer, Shimadzu). The results were expressed as mg gallic acid equivalents mg GAE/L.

The total flavonoid content (TFC) in the vinegars was determined according to the method described by Chen and Chen (2011). The absorbance of the mixture was measured at 510 nm. The catechin was used as a standard and the results were expressed as mg catechin equivalents (mg CE/L).

The free radical scavenging activities of the vinegars were determined with 1,1‐diphenyl‐2‐ picrylhydrazyl (DPPH) according to Lee et al. (1998). The absorbance was measured at 517 nm using a spectrophotometer. The results were expressed as a percentage of free radical scavenging activity (%).

#### 2.4.2 Determination of phenolic composition

According to the method by Pashazadeh et al. (2021), the extraction of phenolic compounds in vinegars was determined. Firstly, 2 mL of the vinegar was shaken with 20 mL of methanol (80%). The mixtures were kept 12 h, filtered, and prepared to detect the phenolic compounds. Shimadzu‐HPLC equipped with a PDA detector and an Inertsil ODS‐3 (5 μm; 4.6 × 250 mm) was used to analyze the compounds as reported by Babiker et al. (2021).

#### 2.4.3 Determination of organic acids

Determination of organic acids was determined by HPLC using an Agilent 1260 Infinity HPLC System equipped with ultraviolet detector set at 210 nm. All fruit vinegars were centrifuged at 10,000 rpm and were filtered via a 0.45‐μm membrane. Then, 20 μL of different vinegar and authentic external standards of organic acids were separately injected into the column. Separation was carried out using a BIORAD Aminex HPX‐87H at 35°C. The mobile phase was 0.005 M H_2_SO_4_ with a flow rate of 0.6 mL/min (Liu et al., 2011).

### 2.5 Effects of marination on meat quality

#### 2.5.1 pH measurements

The pH measurement was carried out with a pH meter (WTW series pH 720) in previously homogenized beef steaks as described by Roudbari et al. (2020). The pH meter was first calibrated with buffer solutions pH 4, 7, and 10. The pH values were determined in triplicate for each sample.

#### 2.5.2 Determination of cooking loss, marinade uptake, drip loss, and yield

Cooking loss (CL), marinade uptake (MU), drip loss (DL), and yield were calculated by measuring the weight changes during marinating and cooking according to the method described by Petracci et al. (2012). The weights of the individual samples were recorded before marination (w1), after marination (w2), after 24 h of storage (w3), and after cooking (w4). The following calculations were made:

$$\text{Marinade uptake}\;\left(\%\right)=\left[\left(w2–\text{w1}\right)/\left(\text{w1}\right)\right]\times 100.$$

$$\text{Drip loss}\;\left(\%\right)=\left[\left(w2–\text{w3}\right)/\left(\text{w2}\right)\right]\times 100.$$

$$\text{Cooking loss}\;\left(\%\right)=\left[\left(w2–\text{w4}\right)/\left(\text{w2}\right)\right]\times 100.$$

$$\text{Yield}\;\left(\%\right)=\left(w4/\text{w1}\right)\times 100.$$

#### 2.5.3 Color measurement

The parameters of lightness (L*), redness (a*), and yellowness (b*) of the samples were measured using a colorimeter (Konica, Minolta CR 400) with illuminant D65, 2° observer angle, diffuse/O mode, 8 mm aperture for illumination. The color measurement was performed perpendicular to the sample surface at five different locations per sample (Hunt et al., 1991).

#### 2.5.4 Scanning electron microscopy (SEM)

The marinated beef was cut using a razor blade to create longitudinal and cross sections of muscle fibers. The prepared beef was coated with a thin layer of gold by using an ion sputter (E‐1010, Hitachi) (Mazaheri Kalahrodi et al., 2021). The microstructural analysis of the prepared beef was scanned using scanning electron microscope (Evo LS 10, ZEISS) at 500 and 1000 magnification.

#### 2.5.5 Textural analyses

The texture profile analysis (TPA) of cooked samples was performed using a texture analyzer (TA‐HD Plus Texture Analyzer) with the twofold compression method (Sengun et al., 2021). A cylindrical plate (diameter of 36 mm) and a 5‐kg load cell were used. Cubic meat pieces (1 × 1 × 1 cm^3^) were prepared from the central parts of the steaks of each treatment. The samples were subjected to double compression as follows: 50% target deformation, recovery time of 5 s, a distance of 5 mm, pretest speed of 1 mm/s, test speed, and posttest speed of 5 mm/s. Results were reported as the mean of at least nine runs for each treatment. The hardness (N), springiness, cohesiveness, chewiness (N), and resilience of the samples were determined.

To determine the Warner–Bratzler shear force (WBSF) and Warner–Bratzler shear energy (WBSE) of marinated and cooked samples, the Warner–Bratzler shear test was performed using a texture analyzer (TA‐HD Plus Texture Analyzer) with a Warner–Bratzler shear attachment consisting of a V‐notch blade. Six cores (1.25 cm in diameter and 2.5 ± 0.3 cm in length) were taken from each sample parallel to the orientation of the muscle fiber. The following settings for WBS texture analysis were made: test mode: compression, pretest speed: 120 mm/min, posttest speed: 600 mm/min, distance: 20 mm. The results were expressed as N for WBSF and N.s for WBSE.

### 2.6 Sensory analysis

The sensory evaluation of the cooked samples was conducted with the hedonic test according to the method given by Modzelewska‐Kapituła et al. (2022). The sensory parameters (9‐like extremely, 1‐dislike extremely) such as color, flavor, odor, and texture were scored by 30 panelists consisting of staff members and graduate students (60% female and 40% male) aged between 20 and 40 from the Selçuk University Food Engineering Department. Briefly, samples were cut into approximately 3‐mm‐thick slices, coded with three‐digit numbers, and served randomly to panelists at room temperature. Water and crackers were provided to cleanse the palate. The same panelists attended three sessions and four samples were presented to each panelist in each session.

### 2.7 Statistical analysis

Results were expressed as means ± SE of three independent (*n* = 3) replicates and a completely randomized factorial design was employed. Data were evaluated using the one‐way ANOVA in Minitab 16.0 statistical program (Minitab Inc.), considering replicate as a random factor. The marination treatment was considered a fixed factor. For sensory data, marination treatment and panelists were considered fixed factors, while tasting order and session number as random effects. Tukey's multiple comparison test was performed to detect significant differences between the treatments, with a significance level of *p* < .05.

## 3 RESULTS AND DISCUSSION

### 3.1 TPC, TFC, and DPPH values of vinegars

Table 2 displays TPC, TFC, and DPPH results in black chokeberry, grape, and hawthorn vinegars. Black chokeberry vinegar had higher TPC, TFC, and DPPH values than grape and hawthorn vinegars (*p* < .05). It has been reported that black chokeberry fruits generally have a higher antioxidant potential than other plant materials (Sidor & Gramza‐Michałowska, 2019). In our previous study, the extract from the pomace of black chokeberry also had higher TPC and TFC values than the pomace extracts of other berries (blackberry, blueberry, and red currant) (Babaoğlu et al., 2022).

**TABLE 2:** The contents of total phenolic (TPC), total flavonoid (TFC), and antioxidant activity (DPPH) in vinegars.

Vinegars	TPC (mg GAE/L)	TFC (mg CE/L)	DPPH (%)
Black chokeberry vinegar	553.43 ± 25.65^a^	153.19 ± 3.14^a^	79.21 ± 5.95^a^
Grape vinegar	344.44 ± 11.32^c^	12.81 ± 0.92^b^	64.73 ± 8.90^b^
Hawthorn vinegar	455.88 ± 16.64^b^	9.57 ± 0.26^b^	49.56 ± 1.57^c^

*Note*: Mean ± SE. Different lowercase superscript letters (a–c) in the same column indicate significant differences (*p* < .05).

### 3.2 Phenolic profile of vinegars

The individual phenolic compounds determined in the fruit vinegars added as ingredients to the marinades are shown in Table 3. The results showed that 12 polyphenols were detected in vinegars. Black chokeberry vinegar contained a higher concentration of catechin (1.17 ± 0.08 mg/L), 3,4‐dihydroxybenzoic acid (0.99 ± 0.13 mg/L) and rutin (0.80 ± 0.23 mg/L). The hawthorn vinegar was rich in gallic acid (3.83 ± 0.80 mg/L), 3,4‐dihydroxybenzoic acid (1.68 ± 0.19 mg/L), catechin (1.66 ± 0.42 mg/L), and rutin (0.46 ± 0.18 mg/L). Gallic acid (5.18 ± 0.45 mg/L), 3,4‐dihydroxybenzoic acid (1.61 ± 0.26 mg/L), catechin (1.07 ± 0.16 mg/L), and rutin (0.52 ± 0.18 mg/L) were the main compounds from the grape vinegar. Similarly, Cosmulescu et al. (2022) found that the gallic acid in grape vinegar was 8.11 mg/L. Kahraman et al. (2022) stated that catechin and gallic acid in grape vinegar were 4.4 and 10.8 μg/L, respectively. Parrilla and Troncoso (1997) observed that gallic acid concentrations in the 92 commercial grape vinegar produced from different wines from the south of Spain ranged from 6.00 to 36.66 mg/L. However, the hawthorn vinegar used in the current study had a lower catechin value than those found by Özdemir et al. (2022). The differences in the contents of phenolics in the literature could be attributed to the various types and different processing stages of vinegar such as fermentation and acidification.

**TABLE 3:** Polyphenolic compositions of vinegars.

Polyphenolic compounds (mg/L)	Black chokeberry vinegar	Grape vinegar	Hawthorn vinegar
Gallic acid	0.26 ± 0.08^b^	5.18 ± 0.45^a^	3.83 ± 0.80^a^
3,4‐dihydroxybenzoic acid	0.99 ± 0.13^b^	1.61 ± 0.26^a^	1.68 ± 0.19^a^
Catechin	1.17 ± 0.28^b^	1.07 ± 0.16^b^	1.66 ± 0.42^a^
Caffeic acid	0.22 ± 0.02^a^	0.07 ± 0.01^b^	0.13 ± 0.03^ab^
Syringic acid	0.12 ± 0.02^a^	0.15 ± 0.05^a^	0.07 ± 0.02^b^
Rutin	0.80 ± 0.23^a^	0.52 ± 0.18^b^	0.46 ± 0.21^b^
p‐Coumaric acid	0.09 ± 0.02^b^	0.06 ± 0.01^b^	0.15 ± 0.06^a^
Ferulic acid	0.21 ± 0.07^a^	0.05 ± 0.01^b^	0.09 ± 0.02^b^
Resveratrol	0.15 ± 0.03	0.15 ± 0.01	0.12 ± 0.02
Quercetin	0.11 ± 0.02^b^	0.23 ± 0.06^a^	0.07 ± 0.02^b^
Cinnamic acid	0.01 ± 0.00^b^	0.01 ± 0.00^b^	0.02 ± 0.01^a^
Kaempferol	0.07 ± 0.02	0.03 ± 0.00	0.06 ± 0.01

*Note*: Mean ± SE. Different lowercase superscript letters (a‐b) in the same row indicate significant differences (*p* < .05).

With respect to the composition of phenolic content, our results showed that catechin was a prominent phenolic compound in black chokeberry vinegar. However, gallic acid was the main phenolic substance in both the grape and hawthorn vinegars. Also, these compounds have known to provide health protective properties including antioxidant, anticancer, and antidiabetic effects (Crozier et al., 2009; Isemura, 2019). In a study that investigated the phenolic compounds of hawthorn vinegar, gallic acid was found to be one of the most important substances (Özdemir et al., 2022). Kelebek et al. (2017) also reported that gallic acid was the main phenolic acid with the highest percentage of the total phenolic acid contents. In the literature, there is no study on the phenolic compounds of black chokeberry vinegar.

### 3.3 Organic acids in vinegars

Vinegars include abundant organic acids, which are the main source of vinegar flavor (Zhu et al., 2016). It has been indicated that organic acids may have a positive effect on meat texture during marinating by reducing thickness and fiber diameter (Roudbari et al., 2020). In this study, the main organic acids of the fruit vinegars and their contents are presented in Table 4. As expected, acetic acid was the most abundant organic acid in the fruit vinegars used in the current study. The highest acetic acid content (23.81 ± 0.15 μg/mL) was determined in the hawthorn vinegar (*p* < .05). Tartaric acid (4.41 ± 0.15 μg/mL) and malic acid (20.04 ± 0.62 μg/mL) were only detected in grape vinegar and citric acid (0.32 ± 0.03 μg/mL) in hawthorn vinegar. The contents of organic acids for grape and hawthorn vinegars obtained in our study were lower than the results of Özdemir et al. (2022) and Lin et al. (2011), respectively. Our lower results of organic acid contents of vinegars could arise from the fruit varieties, growing environments, ripeness stage of fruits, yeast strains, production technique, and fermentation process.

**TABLE 4:** Organic acid compositions of vinegars.

Organic acids (μg/mL)	Black chokeberry vinegar	Grape vinegar	Hawthorn vinegar
Oxalic acid	1.06 ± 0.02^a^	0.04 ± 0.01^b^	0.01 ± 0.00^b^
Citric acid	‐	‐	0.32 ± 0.03
Tartaric acid	‐	4.41 ± 0.21	‐
Malic acid	‐	20.04 ± 0.62	‐
Succinic acid	‐	‐	‐
Lactic acid	1.51 ± 0.22^b^	‐	2.05 ± 0.02^a^
Acetic acid	19.84 ± 0.06^b^	15.22 ± 0.05^c^	23.81 ± 0.05^a^

*Note*: Mean ± SE. Different lowercase superscript letters (a–c) in the same row indicate significant differences (*p* < .05).

### 3.4 pH and weight changes

The effects of marination liquids on cooking loss, drip loss, marinate absorption, and yield values of meat samples are indicated in Table 5. The samples had a cooking loss values ranging from 35.27% to 41.35%, drip loss values ranging from 7.31% to 8.20%. The cooking loss values of meat marinated with different kinds of vinegars were significantly lower than the value of the control group, while no significant effect was determined when comparing drip loss values of control and black chokeberry vinegar group. Usage of BV, GV, and HV significantly affected the marinate absorption values of the meats. The marinate absorption values ranged from 4.25% to 5.36%. Yield values were not affected (*p* > .05) by these liquids. The yield values were in the range of 58.34 to 62.94%. In this line, Sengun et al. (2021) observed that marinate absorption and yield values were between 2.99 and 4.01%, 52.64 and 53.13%, respectively. In a study carried out by Unal et al. (2020), the marinate absorption values of chicken meats increased with a decrease in pH. It was also reported that the addition of acetic acid improved the water‐holding capacity of the samples. In the current study, meat proteins could have been affected by marination with vinegar. This situation is probably due to the low pH values of the meat marinated with vinegars. Therefore, the presence of organic acids in the vinegars can decrease the pH of the marinated meats. As seen in Table 5, the pH values of the samples ranged from 4.68 to 5.80. The pH values of marinated samples were lower than the control group. Likewise, Babikova et al. (2020) and Sengun et al. (2019) observed that acidic solutions decreased the pH values of the meat. If the pH value of the meat is far away from the isoelectric point, the low pH increases the water binding capacity of the meat, which causes a decrease in the cooking loss. This idea is supported by Aktaş et al. (2003) who reported that removing the pH value away from the isoelectric point leads to hold high amount of water in the meat structure. The changes in the pH values of marinated meats could be related to marinade formulation, as demonstrated by Vişan et al. (2021).

**TABLE 5:** pH, cooking loss, marinade uptake, drip loss, yield, and color values of samples.

Analyses	Samples			
	C	BV	GV	HV
pH	5.80 ± 0.08^a^	4.84 ± 0.04^b^	4.68 ± 0.01^b^	4.83 ± 0.01^b^
Cooking loss (CL) (%)	41.35 ± 0.19^a^	38.58 ± 0.10^b^	35.27 ± 0.14^c^	39.33 ± 0.30^b^
Marinade uptake (MU) (%)	4.25 ± 0.32^b^	5.36 ± 0.12^a^	5.31 ± 0.31^a^	5.11 ± 0.09^a^
Drip loss (DL) (%)	7.65 ± 0.15^a^	8.20 ± 0.06^a^	7.31 ± 0.19^b^	7.45 ± 0.15^b^
Yield (%)	62.01 ± 0.04	58.34 ± 0.46	62.94 ± 0.82	59.30 ± 0.17
L*	44.38 ± 0.97^a^	42.42 ± 0.33^ab^	40.61 ± 0.04^b^	43.34 ± 0.12^ab^
a*	7.67 ± 0.24^ab^	8.24 ± 0.21^a^	6.57 ± 0.12^bc^	5.61 ± 0.23^c^
b*	11.88 ± 0.17^a^	8.84 ± 0.06^c^	10.09 ± 0.33^b^	9.14 ± 0.04^bc^

*Note*: Mean ± SE. Different lowercase superscript letters (a–c) in the same row indicate significant differences (*p* < .05). C: Samples marinated with water (vinegar free‐control), BV: Samples marinated with black chokeberry vinegar, GV: Samples marinated with grape vinegar, HV: Samples marinated with hawthorn vinegar. L*: lightness, a*: redness, b*: yellowness.

### 3.5 Color properties

Color properties of the cooked marinated meat samples are indicated in Table 5. Using BV, GV, and HV in meat marination showed a significant effect on L*, a*, and b* values of the samples (*p* < .05). The L* and b* values of the control group (vinegar free‐samples marinated with water) were determined to be higher than other groups. The color of vinegars is colorful, so it is not surprising that the marinade and the meat treated with this marinade are lighter in color. The highest a* values and the lowest b* values were found in samples marinated with black chokeberry vinegar. This situation is due to the redness intensity of black chokeberry. Sengun et al. (2021) observed that the color characteristics of meat were affected by using marinate liquids in meat marination. These researchers also reported that the highest redness values were observed in meat marinated with marinate liquids prepared with blackberry vinegar.

### 3.6 Microstructure

**FIGURE 1:**
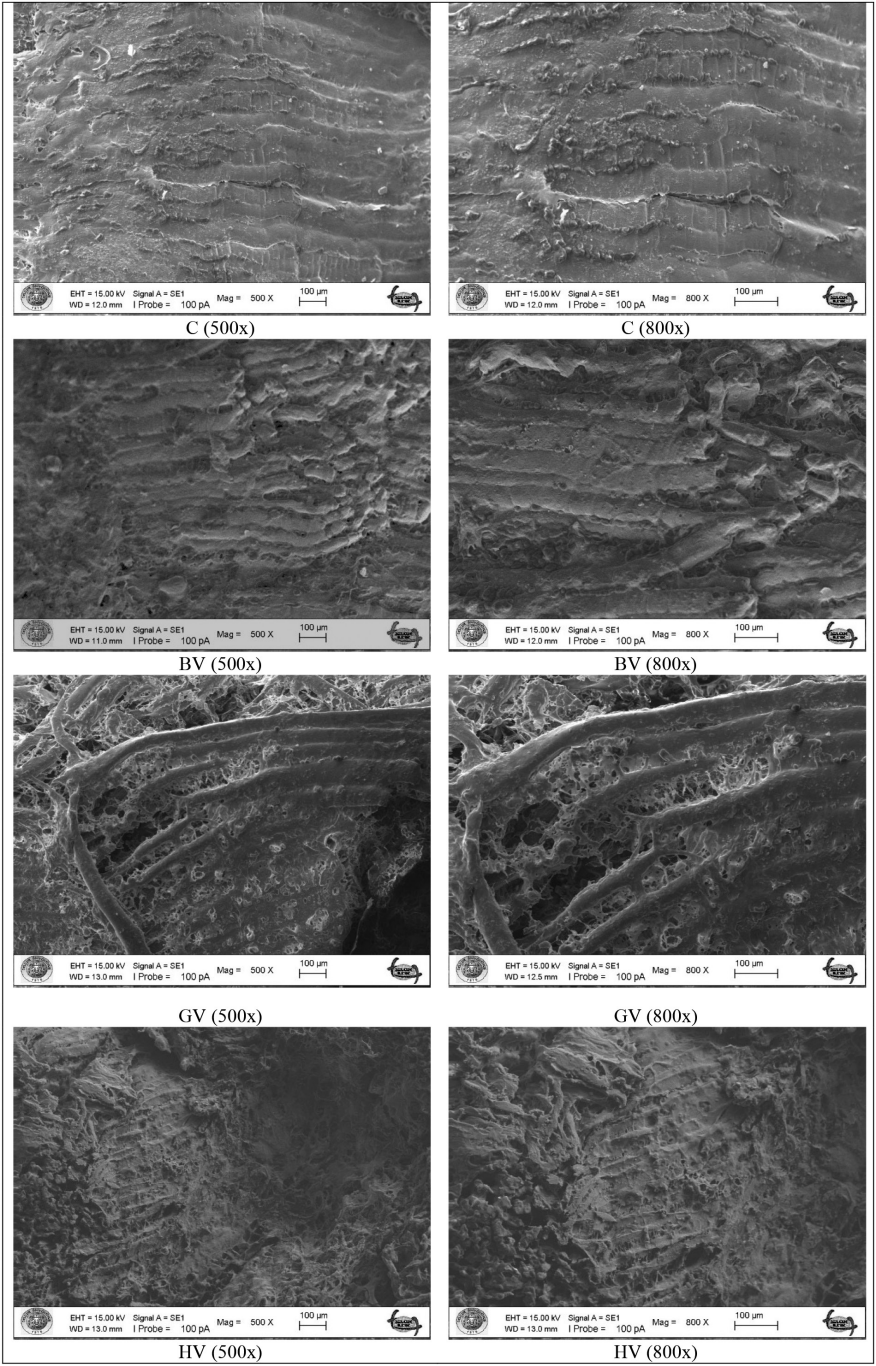
Scanning electron microscopy (SEM) images of control (C), marinated with black chokeberry (BV), grapefruit vinegar (GV), and hawthorn vinegar (HV) at ×500 and ×800 magnification.

Scanning electron microscopy images of beef marinated with black chokeberry vinegar, grape vinegar, and hawthorn vinegar during 24‐h storage are indicated in Figure 1. The fibers from meat marinated with water (control group) were tightly bound to each other. As demonstrated in Figure 1C, SEM images of control group at ×500 and ×800 magnification indicate that there is no gap between beef filaments, which showed meat hardness, whereas SEM images of beef marinated with different kinds of vinegars indicated remarkable deformation and cavities between fibers due to the disruption of connective tissues. As seen in Figure 1 BV, Figure 1 GV, and Figure 1 HV, in marinated samples with both magnifications, the presence of gaps between muscle fibers is clearly visible. These bundle gaps hold the amount of binding water by the muscles as revealed by marinate uptake values in this study. The group in which the effect of vinegar is most pronounced is treated with GV. Similarly, Sunantha and Saroat (2011) reported that the spaces between bundles might be due to the disruption of endomysia collagen and sarcolemma.

A similar SEM image to that noted in the current study was observed by Mazaheri Kalahrodi et al. (2021) for marinated beefsteak chucks using asparagus juice and balsamic vinegar and Unal et al. (2020) for chicken meat treated with fruit juices.

On the other hand, some round‐shaped components were observed in the SEM images. It was thought that they might be salt or black pepper which were used as ingredients of marinade formulation. Therefore, Kim et al. (2013) and Mazaheri Kalahrodi et al. (2021) found similar findings in their studies.

### 3.7 Textural properties

The results of the texture profile and Warner–Bratzler parameters are indicated in Table 6. Marination with different kinds of vinegars showed significant (*p* < .05) effect on hardness value, Warner–Bratzler shear force, and Warner–Bratzler shear energy of the beef samples. The hardness values of the beef marinated with GV and HV were lower than the control and BV group. This situation is probably due to the acidity of the vinegar. Sengun et al. (2021) stated that fruit vinegars could affect the hardness values of meat throughout the marination process due to the organic acid content of vinegars. In their study, marination with rosehip vinegar improved the tenderness of beef. Aziz and Karboune (2018) explained that a possible improvement of textural parameters via marination could be enhanced by the organic acids. Chang et al. (2010) in their research reported that the organic acids were effective in improving the connective meat tissue and a softer texture of beef semitendinosus muscle. Furthermore, Botinestean et al. (2020) demonstrated that malic and citric acid could be applied in tenderizing the beef. These observations were evident in grape vinegar, which was the highest malic acid value, and hawthorn vinegar, which was the highest citric acid value, with a decrease in hardness values of beef, in our study. As revealed by microstructure images (discussed in previous part), marination with different kinds of vinegars affected the meat structure. In this line, Vişan et al. (2021) and Latoch (2020) reported that acidic marinades decreased the pH value which led to the changing of meat structure. They stated that the tenderness was correlated with weakening electrostatic interactions between muscle protein chains. Also, according to Latoch (2020), the tenderness increase is due to the weak. Therefore, Zhang et al. (2021) revealed that moisture content of the meat might be one of the most important factor for the hardness value. In our study, higher marinade uptake values of samples marinated with vinegars resulted in lower hardness value compared to control group.

**TABLE 6:** TPA and Warner–Bratzler results of samples.

Textural analyses	Samples			
	C	BV	GV	HV
TPA parameters				
Hardness (N)	204.66 ± 28.71^a^	198.34 ± 26.74^a^	155.91 ± 13.70^b^	177.84 ± 19.42^ab^
Springiness	0.62 ± 0.04	0.64 ± 0.02	0.65 ± 0.02	0.65 ± 0.02
Cohesiveness	0.54 ± 0.02	0.52 ± 0.02	0.52 ± 0.03	0.50 ± 0.02
Chewiness (N)	64.38 ± 9.11	66.24 ± 7.05	52.51 ± 4.41	57.91 ± 4.87
Resilience	0.16 ± 0.02	0.14 ± 0.01	0.15 ± 0.01	0.13 ± 0.01
Warner–Bratzler parameters				
WBSF (N)	80.81 ± 3.60^a^	70.54 ± 10.17^ab^	53.75 ± 8.33^b^	72.87 ± 5.87^ab^
WBSE (N.s)	371.17 ± 17.59^a^	279.47 ± 34.72^b^	179.62 ± 27.60^c^	339.46 ± 13.09^ab^

*Note*: Mean ± SE. Different lowercase superscript letters (a–c) in the same row indicate significant differences (*p* < .05). C: Samples marinated with water (vinegar free‐control), BV: Samples marinated with black chokeberry vinegar, GV: Samples marinated with grape vinegar, HV: Samples marinated with hawthorn vinegar. WBSF: Warner–Bratzler shear force, WBSE: Warner–Bratzler shear energy.

According to the Warner–Bratzler parameters results, there was a decreasing trend in the samples marinated with vinegars. The lowest WBSF (53.75 N) was found in samples marinated with GV. This was followed by other vinegar treatments, while the highest (80.81 N) WBSF value was determined in the control group. In a study performed by Destefanis et al. (2008), beef tenderness was based on a classification, and it was reported that the WBSF values should be between 32.96 and 42.77 N to obtain a tender beef. In our study, the WBSF values were higher than in Destefanis et al. (2008). This situation is probably due to the Sous vide cooking method applied to their samples. Similar decreases were also reported in WBSF values by previous researchers (Mazaheri Kalahrodi et al., 2021; Naveena et al., 2011; Tkacz et al., 2021).

On the other hand, springiness, cohesiveness, chewiness, and resilience values were not affected (*p* > .05) by using these marinates. Similarly, Botinestean et al. (2016), Latoch (2020), and Sengun et al. (2021) did not observe the effect of marination on springiness and cohesiveness values of the samples.

### 3.8 Sensory scores

**FIGURE 2:**
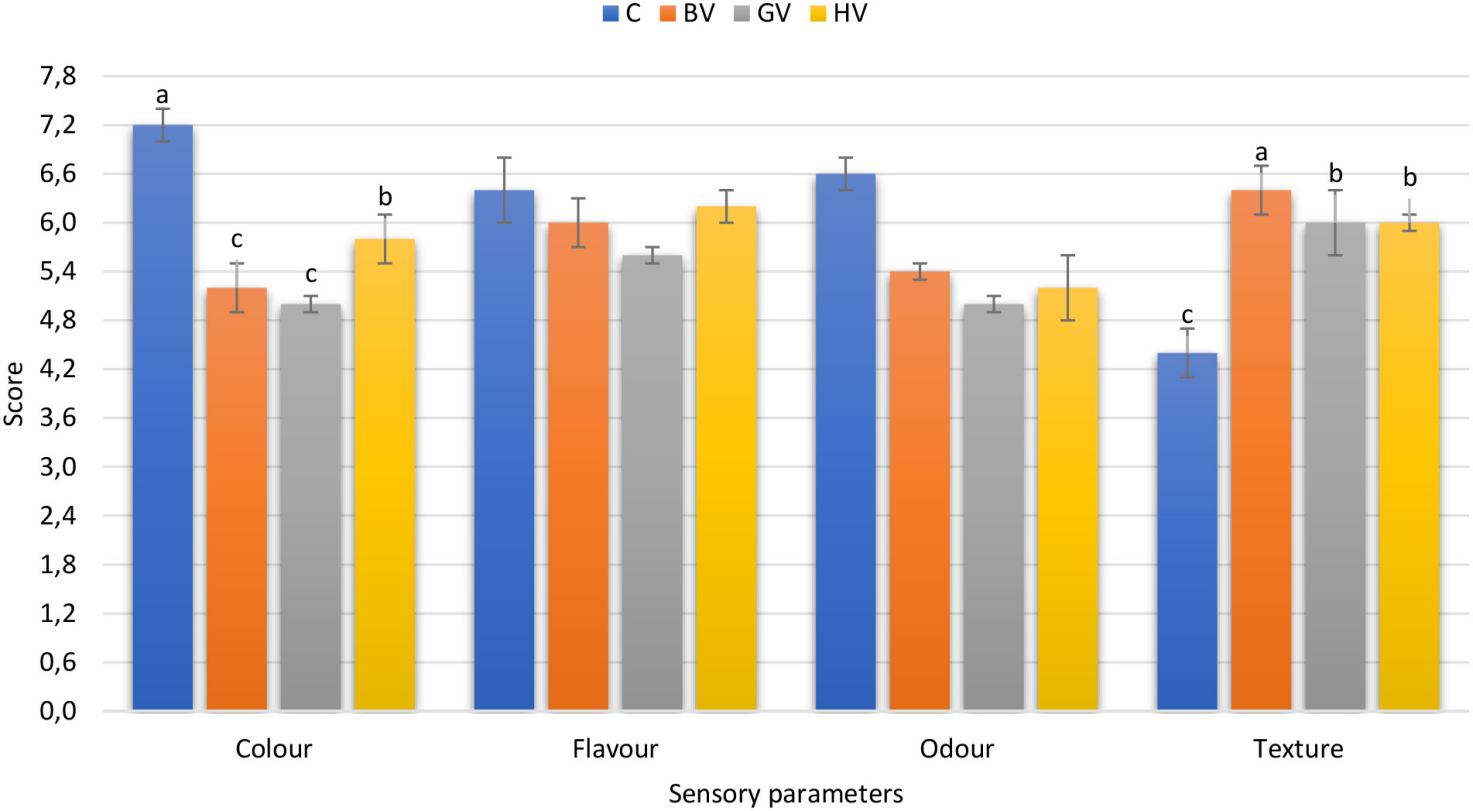
Sensory scores of cooked meat samples marinated with different kinds of fruit vinegars. Bar charts with different letters indicate significant differences between the treatments (a–c) (*p* < .05).

As shown in Figure 2, all sensory quality scores of samples ranged from 4.40 to 7.20. Using different marinades significantly affected the color and texture attributes of beef (*p* < .05). The color property was scored the lowest in samples marinated with vinegars. This was probably due to the characteristic color of vinegar competing with the red‐brown color of the beef under cooking temperature conditions. On the other hand, marinating did not affect the flavor and odor properties of beef negatively. However, texture scores of samples marinated with vinegars were higher compared to the control group. The samples with the highest texture scores were those marinated with black chokeberry vinegar (*p* < .05). More tender and good eating quality was noted in samples marinated with vinegars. This result was in good agreement with the SEM images of meat samples. The obtained results confirm the previous studies revealing that the use of acidic marinates softens the meat texture (Arcanjo et al., 2019; Sengun et al., 2019; Unal et al., 2022).

## 4 CONCLUSION

Marinating with different kinds of vinegars such as black chokeberry (BV), grape (GV), and hawthorn vinegars could be used for improving the textural and quality properties of old *L. lumborum*. The cooking loss values of meat marinated with vinegar were significantly lower than the value of the control group, while usage of BV, GV, and HV significantly affected the marinate absorption values of the meats. Color characteristics were affected by the using these marination liquids. The fruit vinegars were more effective in hardness and WBSF values. The SEM images indicated an important deterioration in muscle fiber. In terms of sensory evaluation, as black chokeberry vinegar improved the texture of the samples, it increased the texture scores. It could be concluded that organic acid containing these fruit vinegars can be natural marination liquid in old cow meat marination for improving consumer acceptability. However, future studies are still needed to increase the sensory properties.

## AUTHOR CONTRIBUTIONS

**Kubra Unal:** Data curation (equal); investigation (equal); methodology (equal); writing – original draft (equal); writing – review and editing (equal). **Ali Samet Babaoğlu:** Data curation (equal); investigation (equal); methodology (equal); writing – original draft (equal); writing – review and editing (equal). **Mustafa Karakaya:** Writing – review and editing (equal).

## FUNDING INFORMATION

This study received no external funding.

## CONFLICT OF INTEREST STATEMENT

The authors declare that they have no conflict of interest.

## ETHICS STATEMENT

This manuscript does not contain any studies with human participants or animals performed by any of the authors.

## Data Availability

The datasets generated during and/or analysed during the current study are available from the corresponding author on reasonable request.
